# The effect of physician remuneration on regional variation in hospital treatments

**DOI:** 10.1007/s10754-015-9164-2

**Published:** 2015-02-01

**Authors:** Rudy Douven, Remco Mocking, Ilaria Mosca

**Affiliations:** 1 CPB Netherlands Bureau for Economic Policy Analysis, P.O. Box 80510, 2508 GM The Hague, The Netherlands; 2 Erasmus University Rotterdam (iBMG), Rotterdam, The Netherlands; 3 Ecorys Netherlands, P.O. Box 4175, 3006 AD Rotterdam, The Netherlands

**Keywords:** Medical practice variations, Financial incentives, Hospital treatments, Remuneration regulations, I110, I180

## Abstract

We study medical practice variations for nine hospital treatments in the Netherlands. Our panel data estimations include various control factors and physician’s role to explain hospital treatments in about 3,000 Dutch zip code regions over the period 2006–2009. In particular, we exploit the physicians’ remuneration difference—fee-for-service (FFS) versus salary—to explain the effect of financial incentives on medical production. We find that utilization rates are higher in geographical areas where more patients are treated by physicians that are paid FFS. This effect is strong for supply sensitive treatments, such as cataracts and tonsillectomies, while we do not find an effect for non-supply sensitive treatments, such as hip fractures.

## Introduction

Regional differences in medical practice variations have long been studied among health services researchers and health economists. Glover (1938) was the first scientist describing the variations in tonsillectomies across geographic areas in the UK. Decades later, the Dartmouth group in the US became widely known and provided evidence for unwarranted regional variations across a wide array of surgical procedures in the US (see for an extensive overview the Darthmouth website).^1^ The seminal paper of Wennberg and Gittelsohn (1973) is considered the first study on the existence of geographic variations of several surgical procedures. Thereafter many more studies appeared that are well documented in Phelps (2000) and Skinner (2012). Both studies provide an in-depth review of the empirical literature on geographical variation.

Much of the empirical evidence stems from the Medicare program in the United States. However in recent years, lots of research on practice variations has been done in different European countries such as England and the Netherlands (Appleby et al. 2011; Westert et al. 2004).

The main challenge within the practice variation literature is to discriminate between warranted and unwarranted variation. Unwarranted variation refers to regional differences that cannot be explained on the basis of illness, strong scientific evidence or the preference of well-informed patients (Fischer et al. 2008). In practice, however, unwarranted variation is extremely difficult to isolate from warranted variation (Mercuri and Gafni 2011).

Wennberg (2010) distinguishes three main categories of care, effective care, price sensitive care and supply sensitive care. In this paper we study the latter category in which differences in payment systems across physicians may influence the clinical decisions of physicians, and thus utilization rates.

That financial incentives play a role in explaining utilization rates is well known (see e.g. Chandra et al. 2012). A famous example is the anecdotal story by Gawande (2009) who argues that physicians in Mc Allen, Texas, are more entrepreneurial than physicians in El Paso, Texas, which results in a dramatic overprovision of several surgical procedures in Mc Allen. Other well-known examples are related to the impact of changes on payment systems on a macro level. For example, in 1983, the Medicare program moved from a retrospective, fee-for-service (FFS) payment to a prospective DRG payment for hospital care. The result was a drop in volume and a large reduction in total hospital days (Hodgkin and Mc Guire 1994). In the Netherlands, the abolishment of hospital budgets in 2001 led to a sharp increase in hospital spending and volume, especially for supply sensitive treatments (Vijsel et al. 2011). Chandra et al. (2012) document the growing literature that studies the impact on utilization rates after exogenous demand shocks or cuts in payments. Many studies find that physicians are likely to respond to financial incentives (see for a systematic review of the literature Chaix-Couturier et al. 2000). However, Chandra et al. (2012) state that the role of fee differences in explaining medical production across patients or geographical areas is unknown. One major reason for a lack of research in this area is that, under Medicare, all physicians receive the same type of payment.

We fill this gap in the literature by studying the effect of different physician remunerations on regional variation in hospital treatments in the Netherlands. Physicians maximize their utility by not only caring about patient benefits but also by caring about their own income and leisure time (McGuire 2000). Depending on the type of payments, a physician may have different choices for these three components, which will subsequently influence patient benefits and utilization rates. Also selection may play a role. Entrepreneurial physicians may put a higher weight on income and will more often choose a FFS than a salary-based payment.

In contrast to Medicare in the US, Dutch physician remunerations differ. Physicians are paid either FFS or salary, and are not constrained by the hospitals in terms of production. We examine variations in eight supply-sensitive surgical procedures and compare the outcomes with hip fractures, a procedure for which unwarranted variation is likely to be small. Moreover, our panel data set covers the totality of the Dutch population over four consecutive years, which allows us to estimate fixed and random effect panel data models.

Our results contribute to the evidence that physicians respond to financial incentives. We show that significantly more patients get treatment in geographical areas with a high percentage of FFS physicians. This effect is strong for highly supply sensitive treatments, such as cataracts and tonsillectomies, while we do not find an effect for weakly supply sensitive treatments, such as hip fractures.

This article is organized as follows: first we describe the “Institutional setting” of the Dutch hospital sector, next we describe our “Data and descriptive statistics”. The “Methods” section explains the estimation procedure and is followed by the “Results” section and “Robustness checks” section. “Conclusions” section concludes.

## Institutional setting

The institutional and regulatory framework of a health care system influence the incentives of both physicians and patients and, hence, the scope for practice variations (Bickerdyke et al. 2002). Several fairly recent institutional factors make the Dutch healthcare system supply sensitive.

Consumers are almost fully insured and there are few incentives for patients to restrain demand.^2^ Except for emergency care, hospital admission occurs only upon GP referral.^3^ Throughout our study period almost all patients could freely choose their preferred hospital.^4^ Patients can thus demand services that provide only a small benefit relative to the costs borne by the insurer. As a result, the Dutch insurance system makes it attractive for physicians to stimulate production, as they know patients will have only minor payment concerns.

The government opted in 2006 to liberalize the delivery of health care, including hospital services. Hospitals are all not-for profit and were historically funded with budgets. In 2001 the strict budgets were replaced by volume-based and open-ended budgets in which “money follows the patients”. This has led to an increase in inpatient admission rates by more than 3 percent per year from 2001 to 2007, while at the same time day care admissions increased by about 9 percent annually (Vijsel et al. 2011). Although the main idea of the liberalization of the health care sector was that health insurers discipline hospitals to deliver care efficiently, an evaluation concluded that this process is still in its early stages (ZonMw 2009).

In 2005 a new hospital payment system, ‘diagnosis treatment combination’ (DTC), was implemented. A DTC relies on an episode-based registration within hospitals. A unique characteristic of the DTC system is the absence of DTC coders, i.e., physicians register DTCs themselves and can change the DTC registration during the treatment (Steinbusch et al. 2007). The DTC system was meant to facilitate the role of insurers as purchasers of care. In 2005, hospitals received a fixed, centrally determined price for initially 90 percent of the DTCs (part A). The remaining 10 percent (part B) was left to volume and price negotiation between health insurers and hospitals. Part B was extended from 10 percent in 2005 to 34 percent in 2009.^5^

Dutch physicians are either self-employed professionals organized by specialty in partnerships (FFS physicians) or they receive a salary from the hospital (Schäfer et al. 2010).^6^ Since the introduction of DTCs in 2005 FFS physicians received a fixed fee for every treatment.^7^ The income earned by FFS physicians is mainly determined by their production. Financial incentives for FFS physicians and hospital management are aligned since the hospital can also increase revenue by treating more patients.

Salaried physicians receive a monthly fixed wage irrespective of their production.^8^ We hypothesize that salaried physicians will put different weights to patient benefits—and therefore utilization rates—than FFS physicians. Since the abolition of fixed hospital budgets in 2001 created room for additional production we hypothesize that medical production and utilization rates will be higher in areas where relatively more patients visit a FFS physician.^9^ Note that this hypothesis is very different from saying that FFS physicians have a higher production level than salaried physicians, which is generally true (see “Data and descriptive statistics” section).

Between 2006 and 2009 the Netherlands had 8 university hospitals and 2 specialty hospitals. General hospitals decreased from 88 in 2006 to 85 in 2009 due to mergers (NZa 2010, 2011). The liberalization of the hospital market led to a concentrated market of general hospitals and a rapid growth of private clinics, often hospital-affiliated. The number of private clinics increased from 37 in 2005 to 129 in 2009. Private clinics offer only part B treatments and accounted for about 6 percent of total production (NZa 2010). The opening of new private clinics or changes in the remuneration schemes or capacity in hospitals creates the variation in the percentage of different types of physicians visited by patients in Dutch zip code areas.

## Data and descriptive statistics

The analysis relies on three data sources: the Dutch Healthcare Authority (NZa) for DTC information and waiting lists, Statistics Netherlands (CBS) for demographic and socio-economic factors, and Dutch Hospital Data (DHD) for information on physicians working in hospitals.

### DTC data and the construction of treatment density

DTC information for the period 2006–2009 was drawn from administrative data collected by the NZa (DTC-informatiesysteem DIS). Table 1 provides a summary of our dataset and description for each treatment: the number of annual DTCs, the patient’s average age, the percentage of men, the number of hospitals and private clinics performing the treatment, and our ex-ante expectation on supply sensitivity.

**Table 1:** Number of treatments, patient and hospital characteristics, and supply-sensitiveness of treatments

Treatment	Specialty	Number of treatments				Average age	Percentage men	Number of hospitals and clinics$^\mathrm{b}$	Ex-ante chance supply sensitiveness
		2006	2007	2008	$2009^\mathrm{a}$				
Cataract	Ophthalmology	134,107	144,874	160,231	145,339	70	39	117	High
Tonsillectomy	Otolaryngology	61,707	62,000	57,007	53,178	10	50	103	High
Hernia	Neurology	49,682	49,180	53,130	52,001	49	51	107	Medium
Varicose veins	Dermatology	37,366	45,526	53,510	56,051	49	15	139	High
Varicose veins	Surgery	26,551	30,721	34,491	33,912	49	25	123	High
Inguinal hernia	Surgery	29,105	30,983	30,415	27,555	48	89	109	Medium
Arthrosis (knee)	Orthopedics	30,561	32,721	34,272	31,016	60	39	111	Medium
Arthrosis (hip)	Orthopedics	21,765	22,155	23,222	21,099	66	32	104	Medium
Hip fracture	Surgery	9,428	9,648	10,141	9,493	76	30	96	Weak

$^\mathrm{a}$ Note that in 2009 the reported number of patients is somewhat lower than in previous years. One explanation is that the dataset for the year 2009 is still incomplete. After closing a DTC providers must send this information to the *DTC-informatiesysteem DIS*. There is no legal instrument to commit providers to deliver DTC’s within a specified time period. This causes some time lags in getting the data

$^\mathrm{b}$ There are 8 university hospitals. The number of general hospitals decreased from 88 in 2006–85 in 2009. Hospitals are obliged to report their DTC data to the NZa but this requirement does not hold for private clinics. In our dataset we have 44 (2006), 57 (2007), 74 (2008) and 78 (2009) unique private clinics while the NZa (2010) reports 57 (2006), 68 (2007), 89 (2008) and 129 (2009) private clinics in the B part. It is not entirely clear how much data we may miss since the NZa (2010) counts also private clinics that perform exclusively treatments we do not consider

The nine treatments are chosen on the basis of recurrence and supply sensitivity. Recurrence is important to obtain enough power for econometric tests. The degree of supply sensitivity is important to check whether our results are in line with ex-ante medical expectations. According to the consulted medical advisors, the treatments (cataract, tonsillectomy and varicose veins, hernia, inguinal hernia, arthrosis) are supply sensitive, and hip fracture is non-supply sensitive.

Our dataset includes about 1.7 million DTCs collected from all Dutch general and university hospitals and 78 private clinics. For each DTC we obtained the four-digit zip code of the patient’s residence as well as the four-digit zip code of the hospital visited by the patient. We used the four-digit zip codes to construct a panel dataset with zip codes as units and years as periods. A DTC is opened at the first physician consult and closed at final examination.^10^ We assign a treatment to the year in which the DTC is opened since about 75 percent of DTCs are completed within the same year. Each hospital diagnosis corresponds to a homogeneous group of unique DTC codes within a medical specialty. Appendix provides further information on the DTC codes.

Roughly 8 percent of our data contains incomplete DTC records. Some hospitals delivered incorrect information, such as wrong or non-existent zip codes, which are crucial for the construction of our panel dataset. We thus deleted a two-digit zip code area when a hospital in that area delivered incorrect zip codes for more than 20 percent of its treatments in a given year.^11^

Our dependent variable, treatment density, is defined as the number of treatments in a four-digit zip code area divided by the population size,^12^ which creates a panel data set of repeated observations for approximately 3,600 four-digit zip code areas for the years 2006–2009. For very small areas, treatment density shows greater variation and we were confronted with missing values and outliers.^13^ According to Diehr et al. (1992) geographical areas should not be too small; we thus excluded all four-digit zip code areas with less than 500 inhabitants, losing 1 percent of the total number of DTC records and about 850 four-digit zip code areas. The final analysis relied on about 3,000 four-digit zip code areas. The descriptive statistics for treatment density are presented in Table 2.

**Table 2:** Descriptive statistics of treatment density

Treatment	Specialty	Average treatment density			
		$2006$	$2007$	$2008$	$2009$
Cataract	Ophthalmology	7.6 (4.4)	8.5 (4.6)	9.5 (5.2)	8.7 (4.8)
Tonsillectomy	Otolaryngology	3.7 (2.1)	3.7 (2.0)	3.3 (1.9)	3.1 (1.9)
Hernia	Neurology	2.8 (1.6)	2.9 (1.6)	3.1 (1.8)	3.1 (2.0)
Varicose veins	Dermatology	2.2 (2.2)	2.7 (2.3)	3.3 (2.8)	3.4 (2.8)
Varicose veins	Surgery	1.6 (1.1)	1.9 (1.3)	2.1 (1.5)	2.1 (1.5)
Inguinal hernia	Surgery	1.8 (1.0)	2.0 (1.1)	1.9 (1.1)	1.7 (1.1)
Arthrosis (knee)	Orthopedics	1.8 (1.3)	2.0 (1.3)	2.1 (1.5)	2.0 (1.3)
Arthrosis (hip)	Orthopedics	1.3 (1.0)	1.4 (1.0)	1.4 (1.1)	1.3 (1.0)
Hip fracture	Surgery	0.6 (0.9)	0.6 (0.8)	0.6 (1.0)	0.5 (0.9)

The figures are the average treatment densities per 1,000 inhabitants of the included four-digit zip code areas in our regressions. The standard deviations are in parentheses

### Control variables

From Statistics Netherlands (CBS) we collected demographic and socio-economic data at four-digit zip code level (see Table 3).^14^

**Table 3:** Descriptive statistics of 4-digit zip code areas

	2006	2007	2008	2009
Total number zip codes	4,007	4,014	4,015	4,019
Number of zip codes included in regressions	2,603	2,640	2,864	2,929
Average population	5,101	5,222	5,020	5,094
Control variables (standard deviation)				
*age0_5 (%)*	6.1 (1.7)	5.9 (1.7)	5.7 (1.7)	5.5 (1.6)
*age5_10*	6.3 (1.6)	6.3 (1.6)	6.4 (1.6)	6.3 (1.6)
*age10_15*	6.3 (1.5)	6.3 (1.5)	6.2 (1.5)	6.2 (1.5)
*age15_20*	6.1 (1.3)	6.2 (1.4)	6.2 (1.3)	6.2 (1.3)
*age20_25*	5.5 (2.7)	5.5 (2.9)	5.5 (2.9)	5.6 (2.9)
*age25_30*	5.5 (2.6)	5.4 (2.6)	5.4 (2.6)	5.4 (2.6)
*age30_35*	6.5 (2.0)	6.1 (2.0)	5.8 (2.0)	5.6 (2.0)
*age35_40*	7.9 (1.6)	7.8 (1.6)	7.6 (1.6)	7.3 (1.6)
*age40_45*	8.1 (1.3)	8.1 (1.3)	8.0 (1.3)	8.0 (1.3)
*age45_50*	7.6 (1.3)	7.7 (1.3)	7.8 (1.3)	7.9 (1.3)
*age50_55*	7.1 (1.4)	7.1 (1.4)	7.2 (1.4)	7.3 (1.4)
*age55_60*	7.3 (1.7)	7.1 (1.6)	6.9 (1.5)	6.9 (1.5)
*age60_65*	5.4 (1.5)	5.9 (1.6)	6.4 (1.7)	6.6 (1.7)
*age65_70*	4.4 (1.4)	4.5 (1.4)	4.6 (1.4)	4.7 (1.4)
*age70_75*	3.6 (1.3)	3.6 (1.3)	3.7 (1.3)	3.8 (1.3)
*age75_80*	2.8 (1.3)	2.9 (1.3)	2.9 (1.3)	3.0 (1.3)
*age80_85*	2.0 (1.2)	2.0 (1.2)	2.0 (1.2)	2.0 (1.2)
*age85_90*	1.0 (0.9)	1.0 (0.9)	1.1 (0.9)	1.2 (0.9)
*age90_95*	0.4 (0.4)	0.4 (0.4)	0.4 (0.4)	0.4 (0.4)
*age95*	0.1 (0.1)	0.1 (0.1)	0.1 (0.2)	0.1 (0.2)
*Men (%)*	49.9 (1.8)	49.9 (1.8)	49.9 (1.8)	49.9 (1.8)
*Mortality (per 1,000)*	7.5 (5.8)	7.4 (5.8)	7.5 (5.6)	n/a
*Westerns (%)*	7.7 (4.9)	7.8 (4.9)	7.8 (4.9)	7.9 (4.9)
*Nonwesterns (%)*	7.1 (10.6)	7.1 (10.7)	7.2 (10.8)	7.4 (10.9)
*Urbanized *	3.7 (1.4)	3.6 (1.4)	3.6 (1.4)	3.6 (1.4)
*social assistance (per 1,000)*	35.4 (37.5)	32.3 (34.8)	29.7 (32.7)	28.9 (31.2)
*Lowincome (%)*	40.5 (5.9)	40.3 (5.8)	n/a	40.2 (6.6)
*Highincome (%)*	20.5 (7.5)	20.7 (7.5)	n/a	19.9 (7.8)
*Working (% of population 15–65)*	71.3 (6.1)	73.4 (5.9)	74.3 (5.8)	n/a
*Selfemployed (% of working people)*	9.2 (4.5)	9.4 (4.5)	9.5 (4.4)	n/a
*distGP (km)*	n/a	1.3 (1.2)	1.4 (1.2)	n/a
*av3GP (number of GP’s within 3 km)*	n/a	7.0 (9.9)	6.8 (9.8)	n/a
*distGPcentre (km)*	n/a	7.3 (5.1)	7.3 (5.1)	n/a
*av20hospital (number of hospitals within 20 km) *	n/a	4.3 (4.1)	4.4 (4.2)	n/a

We included several variables in our analysis that indirectly control for health status.^15^ The first 20 variables in Table 3 reflect the age distribution for 5-year cohorts per four-digit zip code. Next, we include information on gender and social and economic status of the population, the zip codes’ income distribution (three classes of national income distribution: the lowest 40 percent, the upper 20 percent, and the middle 40 percent) and data on the working and self-employed population and people receiving social assistance. We also included four urbanization levels for each zip code area, defined as the number of addresses per square kilometre. Another factor that potentially influences regional health care use is the mortality rate, defined as the number of deceased per 1,000 inhabitants.^16^

The number of treatments in a geographical area may be associated with the availability of health care. For example, if the number of providers in an area increases, travel costs decrease, thereby facilitating access to care. We included the number of hospitals within 20 km as a proxy for hospital availability and three proxies for GP availability, (1) the average distance to the closest GP, (2) the number of GPs within a radius of three kilometres, and (3) the average distance to the closest GP centre. In the Netherlands GPs work as gatekeepers; patients need a referral for hospital admission. For some years data was unavailable (see Table 3). In those cases we used data for adjacent years as a proxy.

We controlled for excess demand with data on average annual waiting times, which we obtained from NZa (Table 4). Data was available for six treatments. For hip fractures waiting time was less relevant since in most cases treatment is emergent. We calculated the weighted average waiting time in a four-digit zip code area by weighing waiting times of all hospitals that patients from a specific zip code visited.

**Table 4:** Average waiting times of two-digit zip code areas

Treatment	Specialty	Average waiting time in weeks			
		$2006$	$2007$	$2008$	$2009$
Cataract	Ophthalmology	6.6 (2.2)	6.4 (2.4)	6.1 (2.0)	4.9 (2.3)
Tonsillectomy	Otolaryngology	5.0 (1.4)	4.7 (1.3)	4.2 (1.2)	3.9(1.3)
Hernia	Neurology	n/a	n/a	n/a	n/a
Varicose veins	Dermatology	n/a	n/a	n/a	n/a
Varicose veins	Surgery	6.8 (2.2)	6.2 (2.3)	5.6 (2.4)	4.4 (2.1)
Inguinal hernia	Surgery	4.6 (1.0)	4.3 (1.2)	4.6 (1.3)	4.3 (1.4)
Arthrosis (knee)	Orthopedics	9.8 (3.6)	8.3 (2.6)	7.8 (1.9)	6.7 (2.2)
Arthrosis (hip)	Orthopedics	8.3 (2.6)	7.7 (2.0)	7.3 (1.8)	6.8 (2.2)
Hip fracture	Surgery	n/a	n/a	n/a	n/a

For Hernia (neurology), Varicose veins (dermatology) and Hip fracture (surgery) we did not include waiting lists in our estimations. The standard deviations are in parentheses

### Physician data

We obtained data on the type of physicians from Dutch Hospital Data (DHD). For almost all individual general and university hospitals we had for each specialty information on their type of remuneration (FFS versus salary). The majority of physicians (about 68 % on average) were paid FFS. For both types of salaried physicians, university hospital (UH) physicians treat on average fewer patients than general hospital (GH) physicians, presumably because they devote more time to education, research, and more complicated treatments. Kruijthof Kruijthof (2005) reports that Dutch FFS physicians have longer working hours, devote more time to patients and have fewer management responsibilities than salaried physicians. One could argue, however, that this may also be the result of patient selection, such as treating a higher proportion of short-stay patients (Wright 2007). Longer working hours may also reflect that FFS physicians put a lower weight on leisure time than salaried physicians.

We constructed the average percentages of physicians visited by patients for each remuneration type (see Table 5).^17^ Interestingly, this approach helped us to provide some extra information on our data limitations, since this allowed us to identify the share of patients, about 15 percent on average and for varicose veins about 30 percent, who visited a physician of an unknown (UN) type. Recall that the majority of UN physicians works in private clinics and is paid FFS. This enables us to approximate the supply sensitivity effect of physicians working in private clinics as well.

**Table 5:** The average percentage of physicians visited by patients, per type of physician (*UH* university hospital, *GH* general hospital, *FFS* fee-for-service, *UN* unknown)

Treatment	Specialty	Average % visited per zip code area															
		$2006$				$2007$				$2008$				$2009$			
		UH	GH	FFS	UN	UH	GH	FFS	UN	UH	GH	FFS	UN	UH	GH	FFS	UN
Cataract	Ophthalmology	6	6	77	12	5	4	77	14	5	5	73	17	5	6	71	18
Tonsillectomy	Otolaryngology	4	5	84	7	4	5	79	11	4	4	78	14	4	4	80	12
Hernia	Neurology	3	15	71	11	4	15	69	12	3	21	60	16	3	17	65	15
Varicose veins	Dermatology	6	9	60	25	5	6	58	31	5	5	58	32	5	5	54	35
Varicose veins	Surgery	3	5	67	25	2	5	61	32	2	5	58	35	1	4	59	35
Inguinal hernia	Surgery	8	6	78	8	7	6	76	11	7	6	74	13	7	5	78	11
Arthrosis (knee)	Orthopedics	4	6	77	13	4	5	75	15	3	5	74	17	3	5	76	16
Arthrosis (hip)	Orthopedics	5	6	81	8	4	5	79	12	4	5	76	14	4	5	80	12
Hip fracture	Surgery	6	7	80	8	6	5	78	11	5	5	76	14	6	4	79	11
Number of 2-digit areas (included in regression)		78				78				84				86			

FFS physicians are present in each year in every zip code area. GH and UH physicians are not always present. In 12 percent (hernia, surgery) to 53 percent (tonsillectomy) of the four-digit zip code areas none of the patients in the corresponding two-digit zip code area visited a GH physician. In 0 percent (cataract) to 41 percent (hip fractures) of the four-digit zip code areas none of the patients in the corresponding two-digit zip code area visited a UH physician. In 45 percent (hip fractures) to 100 percent (varicose veins, surgery) of the two-digit zip code areas, at least one of the patients visited a hospital for which the type of physicians is unknown/unobserved

We defined the average percentages of physicians on the two-digit zip code level. There are about 90 two-digit zip code areas in the Netherlands. By using a more aggregated zip code we obtained a smoother pattern without large outliers. Moreover, defining our supply side variables on the four-digit zip code level was problematic when there are zero patients treated in a certain area. All percentages would then be zero. When one more patient is treated in a certain geographical area, at least one of the percentages becomes positive. This would create positive artificial correlation between treatment density and the supply side variables. The same reasoning holds for the three-digit zip code level. The disadvantage of aggregating to a two-digit zip code level is that some variation across areas is lost. However, Table 6 shows that there remains enough between and within variation of the physician percentages.

**Table 6:** Standard deviations physician percentages

Treatment	Specialty	UH standard deviation			GH standard deviation			FFS standard deviation			UN standard deviation		
		Overall	Between	Within	Overall	Between	Within	Overall	Between	Within	Overall	Between	Within
Cataract	Ophthalmology	9.9	9.5	0.9	15.3	15.5	6.8	28.3	25.3	13.3	22.4	18.2	12.7
Tonsillectomy	Otolaryngology	7.9	7.5	1.4	13.8	13.9	5.3	27.5	23.9	14.0	23.1	18.1	13.9
Hernia	Neurology	9.7	9.2	1.0	28.0	27.7	9.8	34.6	31.9	14.5	22.5	17.8	13.4
Varicose veins	Dermatology	8.4	8.0	1.9	15.3	13.7	6.9	31.3	27.9	14.6	28.9	25.0	14.5
Varicose veins	Surgery	4.4	3.8	1.7	11.1	10.4	5.7	26.8	24.7	11.4	24.9	22.1	11.7
Inguinal hernia	Surgery	9.6	9.1	1.6	14.1	14.1	6.7	26.0	22.6	13.5	21.2	16.6	12.8
Arthrosis (knee)	Orthopedics	7.2	6.7	1.3	12.8	13.1	5.9	24.8	22.2	12.2	20.2	16.5	11.6
Arthrosis (hip)	Orthopedics	8.6	8.1	1.3	12.9	13.7	5.6	25.7	23.0	12.7	21.5	17.1	12.6
Hip fracture	Surgery	13.5	12.8	1.8	12.6	10.9	7.4	28.8	24.9	14.1	23.6	19.2	13.3

## Methods

### Estimation method

We estimated the demand and supply Eq. [1] for each of the nine treatments using OLS, panel data random effects (RE), and panel data fixed effects (FE):

1 $$\begin{aligned} y_{it} =\alpha _i +\gamma _t +\delta _{{\textit{GH}}} p_{{\textit{GH}},it} +\delta _{{\textit{FFS}}} p_{{\textit{FFS}},it} +\delta _{{\textit{UN}}} p_{{\textit{UN}},it} +Z_{it}^{\prime } \beta +\varepsilon _{it} \end{aligned}$$

In Eq. [1], the dependent variable $y_{it}$ is the treatment density in area $i$ in year $t$. The supply side variables $p_{\theta ,it}$ represent the percentage of physicians of type $\theta$ visited by patients from area $i$ in year $t$. Recall that the variables $p_{\theta ,it}$ vary on the two-digit zip code level, while the dependent variable varies on the four-digit zip code level. The vector $Z_{it}$ includes the set of control variables, $\gamma _t$ is a year fixed effect, and $\alpha _i$ is a constant $\alpha$ (OLS) or an individual random (RE) or fixed effect (FE) depending on the estimated specification.

The variables of interest are the supply side variables $p_{\theta , it}$, where we distinguish between the four physician types ($\theta =\hbox {GH, FFS, UN, UH}$). Note that GH and UH are salaried physicians working at general and university hospitals respectively. FFS are FFS physicians working at general hospitals. UN are FFS physicians working for private clinics. We excluded the variable $p_{UH,it}$ since its inclusion would lead to perfect multicollinearity^18^. The estimates of $p_{\theta ,it}$ are thus relative to the base category, which is the percentage of patients visiting a UH physician in area $i$ at year $t$.

If supply sensitivity does not play a role we expect that $\delta _\theta =0$; i.e. the type of physician visited by patients is not related to the treatment density. However, when the remuneration type of the physician does matter we expect the following two hypotheses to hold.

#### **Hypothesis I**

treatment density is higher when patients visit relatively more FFS or UN physicians compared to UH physicians. That is, $\delta _{FFS} >0$ and $\delta _{UN} >0$.

#### **Hypothesis II**

treatment density is higher when patients visit relatively more FFS physicians compared to GH physicians. That is, $\delta _{FFS} >\delta _{GH}$.

Both hypotheses state that FFS and UN physicians have stronger (financial) incentives to treat more patients than UH and GH physicians. We suppose that the size of the effect is stronger for supply sensitive treatments. However, for hip fractures financial incentives are unlikely to play a large role. This leads to the third hypothesis:

#### **Hypothesis III**

Hypotheses I and II do not hold for the treatment of hip fractures.

### Economic effects

The estimated coefficients of interest, $\delta _\theta$, do not provide information about the economic significance of the supply side effects. Therefore we calculated the marginal effects. The marginal effects $\eta _{\theta _1 -\theta _2}$ represent the effect on treatment density of a one percentage point increase in $p_{\theta _1}$, while $p_{\theta _2}$ decreases simultaneously by one percentage point. The marginal effects are measured as a percentage change in treatment density compared to the average treatment density. Formally:

2 $$\begin{aligned} \eta _{\theta _1-\theta _2} =100\% \times \left( {\frac{\hat{\delta }_{\theta _1} -\hat{\delta }_{\theta _2}}{\bar{y}}} \right) \end{aligned}$$

In addition to these baseline estimates, we make several robustness checks. We correct for patients’ cross border mobility and we tackle the low within variation for UH physicians by adding the percentage of UH physicians to the percentage of GH physicians. The rationale behind this is that UH and GH physicians are both paid a fixed salary.

## Results

### Estimation results

Table 7 presents the full estimation results for cataract surgery. For all nine treatments we present the estimated coefficients on our variables of interest $(p_{\theta ,it})$ in Tables 8, 9, and 10.

**Table 7:** Estimation results cataract surgery (t-statistics in parentheses)

	$(1)$	$(2)$	$(3)$	$(4)$
	OLS	OLS	RE	FE
*Percentage_GH*		0.047***	0.048***	0.270***
		(11.8)	(11.8)	(6.7)
*Percentage_FFS*		0.060***	0.065***	0.294***
		(19.1)	(19.2)	(7.4)
*Percentage_UN*		0.061***	0.066***	0.292***
		(17.4)	(17.9)	(7.4)
*Dummy 2007*	0.701***	0.649***	0.648***	0.610***
	(7.9)	(7.4)	(7.5)	(3.6)
*Dummy 2008*	1.447***	1.405***	1.452***	1.599***
	(12.4)	(12.1)	(12.7)	(6.5)
*Dummy 2009*	0.403**	0.408**	0.531***	0.840**
	(3.2)	(3.3)	(4.3)	(2.9)
*Age75_80*	0.837	1.003*	1.074*	1.082
	(1.8)	(2.1)	(2.3)	(1.6)
*Age80_85*	0.978*	1.100*	1.014*	0.752
	(2.0)	(2.3)	(2.1)	(1.1)
*Men*	0.016	$-$0.024	$-$0.020	0.019
	(0.3)	($-$0.4)	($-$0.3)	(0.1)
*Mortality*	$-$0.026	$-$0.029*	$-$0.019	0.002
	($-$1.5)	($-$1.7)	($-$1.2)	(0.1)
*Westerns*	$-$0.072***	$-$0.042***	$-$0.039**	0.042
	($-$5.3)	($-$3.4)	($-$3.2)	(0.5)
*Nonwesterns*	0.026***	0.027***	0.026**	$-$0.142
	(3.1)	(3.3)	(3.2)	($-$1.6)
*Urban1*	0.188	0.009	0.090	0.312
	(0.8)	(0.0)	(0.4)	(0.3)
*Urban2*	0.492*	0.320	0.339	0.141
	(2.3)	(1.7)	(1.7)	(0.1)
*Urban3*	0.344*	0.228	0.206	$-$0.310
	(2.4)	(1.6)	(1.5)	($-$0.9)
*Assistance *	0.001	$-$0.001	$-$0.002	0.006
	(0.2)	($-$0.3)	($-$0.6)	(0.7)
*Working*	0.002	$-$0.010	$-$0.010	0.016
	(0.2)	($-$0.7)	($-$0.7)	(0.3)
*Selfemployed*	$-$0.026	$-$0.017	$-$0.015	$-$0.168
	($-$1.5)	($-$1.0)	($-$0.9)	($-$1.7)
*Lowincome*	$-$0.016	$-$0.020	$-$0.017	$-$0.016
	($-$0.8)	($-$1.1)	($-$1.0)	($-$0.6)
*Highincome*	$-$0.002	$-$0.011	$-$0.012	$-$0.049
	($-$0.2)	($-$0.9)	($-$1.1)	($-$1.6)
*Waitingtime*	$-$0.096***	$-$0.063**	0.001	0.072**
	($-$4.7)	($-$3.1)	(0.1)	(3.0)
*distGP*	$-$0.127**	$-$0.140**	$-$0.144**	0.614
	($-$2.6)	($-$2.9)	($-$2.9)	(1.4)
*av3GP*	$-$0.008	0.001	0.004	0.001
	($-$0.5)	(0.1)	(0.3)	(0.0)
*distGPcentre*	$-$0.024*	$-$0.026*	$-$0.027*	$-$0.115
	($-$2.1)	($-$2.3)	($-$2.4)	($-$0.5)
*av20hospital*	$-$0.025*	$-$0.030*	$-$0.031*	$-$0.578**
	($-$1.8)	($-$2.1)	($-$2.2)	($-$2.9)
*Constant* $^\mathrm{a}$	3.564	$-$14.210	$-$18.190	$-$48.520
	(0.1)	($-$0.3)	($-$0.4)	($-$0.8)
Number of observations	11,036	11,036	11,036	11,036
Number of groups			13,061	13,061
$\hbox {R}^{2}$	0.466	0.482		
$\hbox {R}^{2}$ within			0.083	0.101
$\hbox {R}^{2}$ between			0.642	0.187
$\hbox {R}^{2}$ overall			0.480	0.149

$${*}$$ Significant at $$ {p}<0.05$$;

$${**}$$ significant at $$ {p}<0.01$$;

$${*}{*}{*}$$ significant at $$ {p}<0.001$$

$^\mathrm{a}$ For the fixed effect model, the constant gives the average value of the fixed effects

**Table 8:** OLS estimation results all treatments (t-statistics in parentheses)

	Cataract	Tonsillectomy	Hernia	Varicose veins (dermatology)	Varicose veins (surgery)	Inguinal hernia	Arthrosis (knee)	Arthrosis (hip)	Hip fracture
*percentage_GH*	0.047***	0.010***	0.022***	$-$0.018***	0.028***	$-$0.001	0.023***	0.006***	$-$0.002***
	(11.8)	(3.9)	(12.6)	($-$3.6)	(7.0)	($-$0.6)	(10.9)	(3.7)	($-$2.7)
*percentage_FFS*	0.060***	0.017***	0.029***	$-$0.002	0.029***	0.002	0.020***	0.006***	$-$0.001
	(19.1)	(7.4)	(18.0)	($-$0.4)	(7.7)	(1.5)	(10.5)	(4.0)	($-$1.4)
*percentage_UN*	0.061***	0.019***	0.029***	0.011*	0.035***	0.001	0.021***	0.007***	$-$0.000
	(17.4)	(7.4)	(16.8)	(2.3)	(8.8)	(0.9)	(10.4)	(4.6)	($-$0.5)
Year dummies	Yes	Yes	Yes	Yes	Yes	Yes	Yes	Yes	Yes
Controls	Yes	Yes	Yes	Yes	Yes	Yes	Yes	Yes	Yes
Constant	Yes	Yes	Yes	Yes	Yes	Yes	Yes	Yes	Yes
Number of observations	11,036	11,036	11,036	11,036	11,036	11,036	11,036	11,036	11,036
$\hbox {R}^{2}$	0.482	0.221	0.076	0.126	0.102	0.053	0.110	0.170	0.345

$${*}$$ Significant at $${ p}<0.05$$; $${**}$$ significant at $${ p}<0.01$$; $${***}$$ significant at $$ {p}<0.001$$

**Table 9:** RE estimation results all treatments (t-statistics in parentheses)

	Cataract	Tonsillectomy	Hernia	Varicose veins (dermatology)	Varicose veins (surgery)	Inguinal hernia	Arthrosis (knee)	Arthrosis (hip)	Hip fracture
*percentage_GH*	0.048***	0.011***	0.023***	0.020***	0.023***	$-$0.001	0.019***	0.006***	$-$0.002
	(11.8)	(4.3)	(12.5)	(3.5)	(6.2)	($-$0.5)	(8.4)	(3.9)	($-$2.1)
*percentage_FFS*	0.065***	0.019***	0.030***	0.026***	0.024***	0.002	0.021***	0.006***	$-$0.000
	(19.2)	(8.6)	(17.3)	(4.7)	(6.8)	(1.7)	(10.6)	(4.1)	($-$0.8)
*percentage_UN*	0.066***	0.022***	0.029***	0.037***	0.029***	0.002	0.021***	0.007***	0.000
	(17.9)	(8.9)	(16.2)	(6.7)	(7.8)	(1.4)	(10.2)	(4.7)	(0.1)
Year dummies	Yes	Yes	Yes	Yes	Yes	Yes	Yes	Yes	Yes
Controls	Yes	Yes	Yes	Yes	Yes	Yes	Yes	Yes	Yes
Constant	Yes	Yes	Yes	Yes	Yes	Yes	Yes	Yes	Yes
Number of observations	11,036	11,036	11,036	11,036	11,036	11,036	11,036	11,036	11,036
Number of groups	3,061	3,061	3,061	3,061	3,061	3,061	3,061	3,061	3,061
$\hbox {R}^{2}$ within	0.083	0.045	0.014	0.122	0.036	0.014	0.017	0.006	0.002
$\hbox {R}^{2}$ between	0.642	0.352	0.122	0.118	0.160	0.108	0.172	0.362	0.432
$\hbox {R}^{2}$ overall	0.480	0.220	0.075	0.112	0.100	0.052	0.105	0.170	0.330

$${*}$$  Significant at $${ p}<0.05$$; $${**}$$  significant at $${ p}<0.01$$; $${*\!*\!*}~$$  significant at $${ p}<0.001$$

**Table 10:** FE estimation results all treatments (t-statistics in parentheses)

	Cataract	Tonsillectomy	Hernia	Varicose veins (dermatology)	Varicose veins (surgery)	Inguinal hernia	Arthrosis (knee)	Arthrosis (hip)	Hip fracture
*percentage_GH*	0.270***	0.043***	0.039**	0.117***	0.006	0.027***	0.023*	0.027***	0.001
	(6.7)	(4.2)	(2.7)	(8.5)	(0.7)	(3.5)	(2.5)	(3.5)	(0.2)
*percentage_FFS*	0.294***	0.058***	0.044**	0.114***	0.010	0.030***	0.039***	0.028***	0.002
	(7.4)	(5.8)	(3.1)	(8.4)	(1.1)	(3.9)	(4.5)	(3.6)	(0.6)
*percentage_UN*	0.292***	0.060***	0.044**	0.121***	0.010	0.031***	0.036***	0.029***	0.002
	(7.4)	(6.6)	(3.1)	(9.00)	(1.04)	(4.0)	(4.0)	(3.8)	(0.8)
*year dummies*	Yes	Yes	Yes	Yes	Yes	Yes	Yes	Yes	Yes
*controls*	Yes	Yes	Yes	Yes	Yes	Yes	Yes	Yes	Yes
*constant*	Yes	Yes	Yes	Yes	Yes	Yes	Yes	Yes	Yes
Number of observations	11,036	11,036	11,036	11,036	11,036	11,036	11,036	11,036	11,036
Number of groups	3,061	3,061	3,061	3,061	3,061	3,061	3,061	3,061	3,061
$\hbox {R}^{2}$ within	0.101	0.055	0.022	0.144	0.047	0.024	0.032	0.018	0.011
$\hbox {R}^{2}$ between	0.187	0.095	0.003	0.012	0.020	0.016	0.018	0.020	0.000
$\hbox {R}^{2}$ overall	0.149	0.065	0.000	0.018	0.014	0.011	0.014	0.011	0.001

$${*}$$ Significant at $${ p}<0.05$$; $${**}$$ significant at $${ p}<0.01$$; $${*\!*\!*}$$  significant at $${ p}<0.001$$

Column (1) shows the result of an OLS estimation for Eq. [1] where we excluded the supply side variables $p_{\theta ,it}$. Cataract surgeries occur more frequently in areas with a higher share of older people. This reflects that mostly aged people need cataract surgery. Treatment density is lower in areas with relatively more Western immigrants and higher in areas with relatively more non-Western immigrants. For urbanization we find that the largest effects in the middle categories. Waiting time for cataract surgery has a negative impact on the treatment density, indicating that treatment density is lower in areas with a higher waiting time. The variables related to distance to the GP have a negative impact on treatment density as well. For other treatments, the effect of control variables is potentially very different. For instance, for tonsillectomy we find that treatment density is higher in areas with relatively more people in the age group 0 to 5 years. Since we are interested in the impact of physician remuneration, we do not report the full results here, but we limit ourselves to the coefficients of interest. The regression results for all treatments are available from the authors upon request.

In column (2), (3), and (4) of Tables 7, 8, 9, and 10 we add the physician remuneration variables. In the OLS and RE specifications we observe only small changes in the estimated coefficients for the control variables. In the FE model, however, most control variables are no longer significant because of the low within variation of these explanatory variables.

The physician remuneration variables are positive and highly significant in all three specifications. Treatment density is higher when patients visited relatively more FFS, GH, or UN physicians (compared to the UH type). This finding confirms hypothesis I stating that $\delta _{FFS} >0$ and $\delta _{UN} >0$ for cataract treatments. Note that the estimated coefficients on the variables $p_{\theta ,it}$ are much higher in absolute terms in the FE model.This is related to the fact that the within variation for UH physicians is low (see Table 6).^19^ We postpone the discussion of hypothesis II to the next section where we discuss the economic significance by comparing the coefficients on the variables $p_{\theta ,it}$.

In Tables 8, 9, and 10 we show the estimated coefficients on the physician remuneration variables $p_{\theta ,it}$ for all nine treatments. The coefficients in the tables show that hypothesis I is confirmed in all three models for cataract, tonsillectomy, hernia, and arthrosis (knee and hip). For varicose veins (surgery) hypothesis I is confirmed in both the OLS and the RE model. For varicose veins (dermatology) hypothesis I is confirmed in both the RE and the FE model. For inguinal hernia, hypothesis I is only confirmed in the FE model.

Moreover, we can already conclude that hypothesis I is not confirmed for the treatment of hip fractures in all three models. The coefficients on the variables $p_{\theta ,it}$ are all insignificant in the three models, except for the coefficient on $p_{GH,it}$ in the OLS model. This coefficient is very small and does not have the expected sign. This indicates the the treatment of hip fractures is not related to the type of physician visited by patients and confirms the first part of hypothesis III stating that hypothesis I does not hold for the treatment of hip fractures.

With the Breusch and Pagan Lagrange multiplier test we tested the RE model against the OLS model, favouring the RE model (at a 5 percent significance level) for all nine treatments. A generalized Hausman test, in turn, rejects the RE model in favour of the FE model in all cases^20^. However, we continue to present OLS and RE estimates for reasons of comparison.^21^

### Economic importance

Table 11 presents the marginal effects $\eta _{\theta _1 -\theta _2}$. Since the FE model is the preferred specification we focus on the results in columns (3), (6), and (9) of Table 11.

**Table 11:** Marginal effects main model

Treatment	Specialty	$\eta _{{\textit{FFS}}-{\textit{UH}}}$			$\eta _{{\textit{FFS}}-{\textit{GH}}}$			$\eta _{{\textit{FFS}}-{\textit{UN}}}$		
		$(1)$	$(2)$	$(3)$	$(4)$	$(5)$	$(6)$	$(7)$	$(8)$	$(9)$
		OLS	RE	FE	OLS	RE	FE	OLS	RE	FE
*Dependent variable: 4-digit treatment density (N=11,036)*										
Cataract	Ophthalmology	0.70***	0.75***	3.41***	0.15***	0.19***	0.27***	$-$0.01	$-$0.01	0.01
Tonsillectomy	Otolaryngology	0.50***	0.56***	1.67***	0.20***	0.24***	0.42***	$-$0.06*	$-$0.07**	$-$0.07*
Hernia	Neurology	0.98***	1.01***	1.48**	0.24***	0.22***	0.17**	0.01	0.02	0.00
Varicose veins	Dermatology	$-$0.07	0.88***	3.90***	0.55***	0.21***	$-$0.11	$-$0.46***	$-$0.38***	$-$0.26***
Varicose veins	Surgery	1.52***	1.24***	0.52	0.07	0.07	0.19	$-$0.31***	$-$0.23***	0.03
Inguinal hernia	Surgery	0.10	0.12	1.61***	0.15**	0.16**	0.15	0.03	0.02	$-$0.04
Arthrosis (knee)	Orthopedics	1.00***	1.07***	1.96***	$-$0.19**	0.09	0.81***	$-$0.07	$-$0.01	0.11*
Arthrosis (hip)	Orthopedics	0.42***	0.43***	2.04***	$-$0.03	$-$0.02	0.05	$-$0.07*	$-$0.07*	$-$0.05
Hip fracture	Surgery	$-$0.14	$-$0.07	0.31	0.24*	0.23*	0.18	$-$0.09	$-$0.08	$-$0.08

$${*}$$ Significant difference at $${ p}<0.05$$; $${**}$$ significant difference at $${ p}<0.01$$; $${*\!*\!*}$$ significant difference at $${ p}<0.001$$ (based on robust standard errors)

The results in column (3) (6), and (9) of Table 11 show the economic importance of the effects. For example, for cataract surgery we find that treatment density increases by 3.4 percent (compared to the average) when the percentage of patients visiting a FFS-physician increases by one percent and the percentage of patients visiting a UH-physician decreases by one percent.^22^ Column (6) of Table 11 shows that this number is 0.27 percent when the percentage of patients visiting a FFS-physician increases by one percent and the percentage of patients visiting a GH-physician decreases simultaneously by one percent. We observe hardly any difference between FFS and UN-physicians for cataract surgery.

Hypothesis II, stating that $\delta _{FFS} >\delta _{GH}$, is confirmed for cataract surgery, tonsillectomy, hernia, and the treatment of arthrosis (knee) in the FE model (column 6). For these treatments the estimated marginal effect is in the range of 0.17 percent (hernia) to 0.81 percent (arthrosis–knee). For the other treatments we find insignificant results, indicating that there is no difference between FFS and GH physicians. We conclude that for four treatments (cataract, tonsillectomy, hernia, and knee arthosis) practice variations can be explained by differences in the remuneration among physicians.

Besides that, it is interesting to investigate how treatment density relates to the percentage of patients visiting a UN physician. Column (9) of Table 11 shows that UN physicians, who mainly work in private clinics, perform similarly to FFS physicians.^23^ However, we find for the treatment of varicose veins (dermatology) that treatment density is significantly higher in areas where patients visit relatively more frequently a UN physician rather than a FFS physician (see column (9) in Table 11). This suggests that for varicose veins private clinics have increased the number of treatments and that they may have created additional demand.

We also confirm hypothesis III. As already shown in the previous section, none of the physician remuneration variables $p_{\theta , it}$ has a significant impact on the treatment density of hip fractures. Moreover, we find no significant differences between the percentage of FFS and GH physicians in the FE model. In other words, practice variations in hip fractures is not related to the remuneration of the physicians in our preferred specification. In the OLS and RE model, hypothesis II is confirmed for hip fractures. This in contrast to hypothesis III. However, if we take a closer look at the coefficients we see that this results from the fact that the coefficient on $p_{GH,it}$ is negative and significant (almost significant in RE) while the coefficient on $p_{FFS,it}$ is close to zero and not significant.We conclude that for hip fractures this difference is not likely to be related to financial incentives of physicians.

## Robustness checks

### Excluding border areas

Unfortunately, we have no information on cross border mobility. Some patients go abroad for treatment. Cross-border health care costs represent about 1.2 percent of the total health care costs in the Netherlands. This could influence our results if treatment densities are measured incorrectly in border areas. For this reason, we re-run our regressions excluding the 26 two-digit zip code areas that are adjacent to the Belgian and German border. This reduces the total number of observations by about 30 percent (to 7,029 nobs). Table 12 shows the marginal effects resulting from the analysis without border areas. The results are highly comparable to our previous findings presented in Table 11.

**Table 12:** Robustness check border areas

Treatment	Specialty	$\eta _{{\textit{FFS}}-{\textit{UH}}}$			$\eta _{{\textit{FFS}}-{\textit{GH}}}$			$\eta _{{\textit{FFS}}-{\textit{UN}}}$		
		$(1)$	$(2)$	$(3)$	$(4)$	$(5)$	$(6)$	$(7)$	$(8)$	$(9)$
		OLS	RE	FE	OLS	RE	FE	OLS	RE	FE
*Dependent variable: 4-digit treatment density (N=7,926)*										
Cataract	Ophthalmology	1.03***	1.06***	2.41***	0.16***	0.18***	0.23**	$-$0.06	$-$0.03	0.06
Tonsillectomy	Otolaryngology	0.39***	0.50***	1.98***	0.20***	0.23***	0.38**	$-$0.05	$-$0.02	0.03
Hernia	Neurology	2.09***	2.23***	2.93***	0.16***	0.14***	0.09	0.03	0.06	0.07
Varicose veins	Dermatology	0.65***	1.47***	3.84***	0.32***	0.01	$-$0.09	$-$0.68***	$-$0.49***	$-$0.32***
Varicose veins	Surgery	2.36***	1.86***	0.31	0.00	0.01	0.15	$-$0.49***	$-$0.37***	0.01
Inguinal hernia	Surgery	0.37**	0.42**	1.28**	0.10*	0.12**	0.18	0.11**	0.10*	0.05
Arthrosis (knee)	Orthopedics	1.56***	1.64***	1.62**	$-$0.30***	$-$0.02	0.85***	$-$0.06	0.00	0.08
Arthrosis (hip)	Orthopedics	0.71**	0.75**	1.22	$-$0.09	$-$0.09	$-$0.10	$-$0.07	$-$0.06	0.02
Hip fracture	Surgery	0.29	0.24	0.11	0.29**	0.27*	0.18	$-$0.05	$-$0.01	$-$0.03

$${*}$$ Significant difference at $${ p}<0.05$$; $${**}$$ significant difference at $${ p} < 0.01$$; $${*\!*\!*}$$ significant difference at $${ p} < 0.001$$ (based on robust standard errors)

### Low within variation for university hospitals

Table 6 shows that the within variation of the percentage of patients visiting a university hospital is low. Indeed, almost no changes took place on the market of university hospitals during our sample period. For the other group of hospitals we observe that few hospitals exited and new hospital entered the market. Moreover, for a small number of hospitals we observe changes in remuneration schemes of physicians during the sample period. The low within variation for UH physicians enhances identification problems in the FE model because identification is solely based on this variation. For example, the estimated marginal effects $\eta _{FFS-UH}$ are much higher in the FE model than in the other models. To circumvent this problem we add the percentage of UH physicians to the percentage of GH physicians. The rationale behind this is that UH and GH physicians are both paid a fixed salary.^24^ Table 13 presents the results, which are in line with our previous findings. The marginal effects $\eta _{FFS-(GH+UH)}$ are now a weighted average of the former two marginal effects $\eta _{FFS-UH}$ and $\eta _{FFS-GH}$ presented in Table 11. The marginal effects $\eta _{FFS-UN}$ do not change very much compared to the findings in Table 11.

**Table 13:** Robustness check GH and UH together

Treatment	Specialty	$\eta _{{\textit{FFS}}-({\textit{GH}}+{\textit{UH}})}$			$\eta _{{\textit{FFS}}-{\textit{UN}}}$		
		$(1)$	$(2)$	$(3)$	$(4)$	$(5)$	$(6)$
		OLS	RE	FE	OLS	RE	FE
Cataract	Ophthalmology	0.32***	0.33***	0.40***	0.00	0.00	0.01
Tonsillectomy	Otolaryngology	0.28***	0.31***	0.51***	$-$0.05*	$-$0.06**	$-$0.08*
Hernia	Neurology	0.31***	0.28***	0.18**	0.02	0.03	0.00
Varicose veins	Dermatology	0.41***	0.31***	0.21**	$-$0.45***	$-$0.38***	$-$0.28***
Varicose veins	Surgery	0.29***	0.21***	0.21	$-$0.27***	$-$0.21***	0.02
Inguinal hernia	Surgery	0.13***	0.14***	0.24**	0.03	0.02	$-$0.06
Arthrosis (knee)	Orthopedics	0.12*	0.28***	0.87***	$-$0.05	0.01	0.11*
Arthrosis (hip)	Orthopedics	0.12*	0.11*	0.17	$-$0.07*	$-$0.07*	$-$0.06
Hip fracture	Surgery	0.04	0.13	0.19	$-$0.09	$-$0.08	$-$0.08

$${*}$$ Significant difference at $${ p} < 0.05$$; $${**}$$ significant difference at $${ p} < 0.01$$; $${*\!*\!*}$$ significant difference at $${ p} < 0.001$$ (based on robust standard errors)

## Conclusions

The Dutch government liberalized the provision of hospital services in 2001 by payment systems that were to a large extent volume-based and open-ended such that “money follows the patients”. In combination with patients facing limited cost-sharing and free hospital choice these factors led to a strong growth in hospital production.

We showed that physicians respond to financial incentives and these effects appear to be stronger for supply sensitive treatments. We found that utilization rates are higher in areas where more patients are treated by FFS physicians. For example, if patients visit one percent more FFS physicians instead of salaried physicians, then the number of cataract treatments in that area increases by about 0.27 percent. For treatments of varicose veins we find that physicians working in private clinics treat even more patients than physicians working in general hospitals that are paid fee for service. This may suggest a selection effect. Entrepreneurial physicians will put a high weight on income in their utility function, and are therefore more likely to be working for private clinics.

We could identify this effect by using exogenous variation for groups of physicians that face different financial incentives. This variation stems from physicians, hospitals and clinics entering and leaving the market, and from changes in physician remuneration in some hospitals during the sample period. Moreover, the validity of our results is confirmed by performing a similar analysis for the treatment of hip fractures. In line with our expectations, we find that the hip fracture treatment density is not related to physician remuneration.

In this study we were not able to analyse the evolution of the utilization rates before 2005, when the DTC system was introduced in the Netherlands, due to a lack of data and a different registration of medical conditions. We cannot thus reject the hypothesis that an uneven treatment intensity, in the years preceding the DTC implementation, could explain the differences found in the study.

There are many directions for further research. Other supply factors, such as the number of physicians, the degree of concentration of physicians in hospitals, differences in physician practices or prices of treatments, may be important as well for explaining practice variations. It would also add value to have some information on quality of care. Although we find variation between FFS physicians and salaried physicians we cannot infer that we might be witnessing poor quality of health care. Also, adding more control variables, such as the health status of patients, could improve the results.

From a policy point of view it is hard to say whether it is preferable to have more or less physicians on FFS. Our results indicate that allowing FFS physicians and private clinics on the market can be a mixed blessing. On the one hand, these physicians may treat individual patients more efficiently, but on the other hand, they may treat too many patients. If the government believes there exists “overtreatment” then replacing FFS physicians by salaried physicians could be a sensible policy option. However, to answer this question properly we need more information about the possible differences between FFS, UN physicians and salaried physicians. Information about the quality of treatments will help policymakers to make a better decision on having more or less FFS physicians. For example, if it turns out that FFS and UN physicians are delivering better quality of care than salaried physicians then this could also be seen as an argument to support more FFS physicians.

Our finding that supply side factors are related to practice variations may also have important policy implications for supply and demand side regulation. First, if hospital management suspect too much production then they could adjust their contracts with physicians. For example, regressive tariffs could be introduced to soften production. This strategy, however, will be difficult to realize since hospital income depends upon production. Second, the option of being a FFS physician could be made less attractive. This is currently politically debated in the Netherlands. Third, insurers could address unwarranted practice variations by benchmarking hospitals and using managed care activities to discipline physicians. Fourth, unwarranted practice variations can be mitigated by increasing cost-sharing arrangements for consumers regarding those supply sensitive treatments.

## Appendix

Description of DTC codes. See Table 14.

**Table 14:** Description of DTC-codes

Treatment	Specialty & specialty code	Dutch DTC codes	Description
Cataract	Ophthalmology; 301	X1000554003Y	Cataract treatments can essentially be divided into three categories, day treatment, inpatient treatment and outpatient treatment. Dutch medical advisors consider cataract treatments as highly supply sensitive
		X $=$ 1, 2	
		Y $=$ 1, 2, 3, 6	
Tonsillectomy	Otolaryngology; 302	X1000052021Y	Tonsillectomy can essentially be divided into three categories, day treatment, inpatient treatment and outpatient. Dutch medical advisors consider treatments for tonsillectomy as highly supply sensitive
		X $=$ 1, 2	
		Y $=$ 1, 2, 3, 6	
Hernia	Neurology; 330	X100120301YZ	Treatment for hernia within neurology is conservative, i.e. it avoids radical medical therapeutic measures or operative procedures. Dutch medical advisors consider treatments for hernia as supply sensitive
		X $=$ 1, 2	
		Y $=$ 1, 2, 3	
		Z $=$ 1, 2, 3	
Varicose veins	Dermatology; 310	X100002400YZ	Treatment for varicose veins can be: laser treatment to destroy the vein, sclerotherapy to close off the vein, and surgery to tie off or remove the vein. These treatments can be further classified in day treatment versus inpatient/outpatient treatments. These latter are used for the most invasive operations. Dutch medical advisors consider treatments for varicose veins as highly supply sensitive
		X $=$ 1, 2	
		Y $=$ 3, 4, 5, 8, 9	
		Z $=$ 1, 2, 3	
Varicose veins	Surgery; 303	110004230X0Y	Treatment for varicose veins can be: laser treatment to destroy the vein, sclerotherapy to close off the vein, and surgery to tie off or remove the vein. These treatments can be further classified in day treatment versus inpatient/outpatient treatments. These latter are used for the most invasive operations. Dutch medical advisors consider treatments for varicose veins as highly supply sensitive
		X $=$ 2, 4	
		Y $=$ 1, 2, 3, 6	
Inguinal hernia	Surgery; 303	110001210X0Y	Inguinal hernia can be treated with an endoscopic/minimal invasive surgery—usually for relatively easier cases—or with open/classic surgery, which is more invasive. Dutch medical advisors consider treatments for inguinal hernia as supply sensitive
		X $=$2, 3, 4, 5	
		Y $=$1, 2, 3	
Arthrosis (knee)	Orthopedics; 305	X100180102YZ	Arthrosis of the knee can be treated with or without intervention/operation. Also, the operation can include prosthesis or not. Dutch medical advisors consider treatments for arthrosis (knee) as supply sensitive
		X $=$1, 2	
		Y $=$1, 2	
		Z $=$1, 2, 3, 6	
Arthrosis (hip)	Orthopedics; 305	X100170102YZ	Arthrosis of the hip can be treated with or without intervention/operation. Also, the operation can include prosthesis or not. Dutch medical advisors consider treatments for arthrosis (hip) as supply sensitive
		X $=$1, 2	
		Y $=$1, 2	
		Z $=$1, 2, 3, 6	
Hip fracture	Surgery; 303	110002180X03	Most hip fractures are treated by orthopedic surgery, which involves implanting an orthosis. If operative treatment is refused or the risks of surgery are considered to be too high the main emphasis of treatment is on pain relief. Dutch medical advisors consider treatments for hip fractures as weakly supply sensitive
		X $=$2, 4	

## Footnotes

1
http://www.dartmouthatlas.org/.

2 Dutch citizens pay only 5 percent of their health care expenditures out-of-pocket, one of the lowest contributions of EU member states. During the sample period the annual mandatory deductible for basic benefit package was €150 for adults (€0 for children). Adults could choose an additional voluntary deductible up to €500, but only about 5 percent of the population did (NZa 2011).

3 About 63 percent of the patients in Dutch hospitals are referred by a GP. This figure used to be quite stable but declined to 57.7 percent in 2008 and 60.6 percent in 2009, suggesting that physicians increased the number of referrals to other physicians (or to themselves) within hospitals.

4 The health care sector in the Netherlands was fundamentally reformed in 2006 with the introduction of regulated competition . The rationale behind the reform is to stimulate efficiency and curb health care costs while safeguarding governmental objectives such as affordability, quality, and accessibility. From 2006–2009 one small insurer started to experiment with selective contracting of hospitals.

5 Part B was expanded to 70 percent in 2012.

6 Physicians working at university hospitals are salaried. When it comes to general hospitals, physicians can either receive a salary or FFS.

7 A DTC can be characterized as a bundle of services. FFS physicians were present during the budgeting system before the year 2000, but this caused tension because the hospital management tried to restrict their activities (Kruijthof 2005).

8 Although the hospital management may stimulate salaried physicians to increase production, for example by providing bonusses.

9 The in-patient admission rates increased by more than 3 percent per year from 2001 to 2007, while at the same time day care admissions increased by about 9 percent annually (Vijsel et al. 2011). Prior to 2008 medical specialists received a lump sum (fixed budget) payment in part A of hospital care. In each hospital the lump sum was divided among specialists according to past production and fees. As the lump sum was a fixed amount of money, there were no incentives for specialists to increase the production. The lump sum ceased to exist in 2008. Since then medical specialists face the same financial incentives in part A and part B of hospital care.

10 DTCs formally have a maximum length of 365 days. For chronic patients DTCs are automatically closed after a one year-period and reopened thereafter. Steinbusch et al. (2007) provide more information about DTCs and their relation to DRGs.

11 Especially in 2006 and 2008 many hospitals often used the non-existent zip code “1,000” for all treatments. During 2006–2009 36 hospitals had non-existent zip codes for more than 20 percent of their DTCs. Computationally, we followed a similar procedure for all nine treatments and deleted about 400 four-digit zip code areas for which the first two digits were the same as the hospitals’.

12 The number of inhabitants per four-digit zip code area is obtained from CBS (see “Control variables” section).

13 The missing values refer to our explanatory variables obtained from CBS (“Control variables” section).

14 The information is freely available at www.cbs.nl.

15 Variables directly related to health status are not necessarily preferable in explaining regional variation. Inhabitants are more likely to receive treatment if their physician treats them more intensively. This bias may make patients in high treatment areas appear sicker than they actually are.

16 Mortality rate can be viewed as an outcome indicator of health care performances. This is however less relevant for our analysis since our treatments are in general not life-threatening. As an extra check we ran all regressions excluding the mortality rate, but this did not influence our results.

17 For example, if half of the patients in a two-digit area are treated by a university hospitals and the other half by hospitals with only FFS physicians then the FFS- and UH-percentage are both 50 percent and the GH- and UN- percentage are both 0 percent. The average percentages of physicians is also influenced by the fact that in a few number of hospitals the type of renumeration changed during the sample period.

18 Since $$\sum _\theta p_{\theta ,it}=1$$.

19 The low within variation for UH physicians makes it difficult to identify the impact of the different physician remuneration schemes *relative *to the base category of UH physicians. Note, however, that we are still able to identify differences between the other types of physicians (GH, FFS, and UN), because within variation is much higher for these types of physicians.

20 We performed this test with Stata, using the *xtoverid* command (see Schaffer and Stillman 2010).

21 Note, that the OLS and RE estimations provide more stable results and confirm our three hypotheses as well.

22 The marginal effects show the impact on average treatment density. The impact of a 1 percent increase in the percentage of FFS physicians visited and a 1 percent decrease in the percentage of GH physicians visited is a 0.27 percent increase of the average treatment density. Since population stays the same, this is equivalent to a 0.27 percent increase in the number of cataract treatments. The total number of treatment over the period 2006–2009 was just above 580,000 (Tables 1, 2). This would mean that about 1,600 extra cataract treatments would have been performed, if patients had visisted 1 percent more FFS and 1 percent less GH physicians over the period 2006–2009.

23 Note that the individual coefficients $\delta _{UN}$ in Table 10 are all positive and significant, except for varicose veins (surgery) and hip fractures, indicating that the number of treatments is on average higher when patients visited relatively more UN physicians compared to UH physicians.

24 It should be stressed, however, that the incentives for UH and GH physicians are not identical as explained in “Data and descriptive statistics” section.
